# ICT and AIE Characteristics Two Cyano-Functionalized Probes and Their Photophysical Properties, DFT Calculations, Cytotoxicity, and Cell Imaging Applications

**DOI:** 10.3390/molecules25030585

**Published:** 2020-01-29

**Authors:** Arup Tarai, Meina Huang, Pintu Das, Wenhui Pan, Jianguo Zhang, Zhenyu Gu, Wei Yan, Junle Qu, Zhigang Yang

**Affiliations:** Center for Biomedical Photonics, Key Laboratory of Optoelectronic Devices and Systems of Ministry of Education and Guangdong Province, College of Physics and Optoelectronic Engineering, Shenzhen University, Shenzhen 518060, China; aruptarai@gmail.com (A.T.); mernahuang2016@126.com (M.H.); pintuchem@gmail.com (P.D.); panwenhui2017@email.szu.edu.cn (W.P.); jguo_zhang@163.com (J.Z.); zheny_g@163.com (Z.G.); weiyan@szu.edu.cn (W.Y.)

**Keywords:** aggregation, charge-transfer, emission, solvatochromism, cytotoxicity, cell imaging

## Abstract

Two probes, AIE-1 and AIE-2, were synthesized to investigate the effect of substitutional functional group on aggregation (aggregation-caused quenching (ACQ) or aggregation-induced emission (AIE)) and intramolecular charge transfer (ICT) behavior as well as on the cell imaging aspect. The yellow-color non-substituted probe AIE-1 showed weak charge-transfer absorption and an emission band at 377 nm and 432 nm, whereas the yellowish-orange color substituted probe AIE-2 showed a strong charge-transfer absorption and an emission band at 424 nm and 477 nm in THF solvent. The UV-Vis studies of AIE-1 and AIE-2 in THF and THF with different water fractions showed huge absorption changes in AIE-2 with high water fractions due to its strong aggregation behavior, but no such noticeable absorption changes were observed for AIE-1. Interestingly, the fluorescence intensity of AIE-1 at 432 nm gradually decreased with increasing water fractions and became almost non-emissive at 90% water. However, the monomer-type emission of AIE-2 at 477 nm was shifted to 584 nm with a 6-fold increase in fluorescence intensity in THF-H_2_O (1:9, *v/v*) solvent mixtures due to the restriction of intramolecular rotation on aggregation in high water fractions. This result indicates that the probe AIE-1 shows ACQ and probe AIE-2 shows AIE behaviors in THF-H_2_O solvent mixtures. Furthermore, the emission spectra of AIE-1 and AIE-2 were carried out in different solvent and with different concentrations to see the solvent- or concentration-dependent aggregation behavior. Scanning electron microscope (SEM) and dynamic light scattering (DLS) experiments were also conducted to assess the morphology and particle size of two probes before and after aggregation. Both of the probes, AIE-1 and AIE-2, showed less toxicity on HeLa cells and were suitable for cell imaging studies. Density functional theory (DFT) calculation was also carried out to confirm the ICT process from an electron-rich indole moiety to an electron-deficient cyano-phenyl ring of AIE-1 or AIE-2.

## 1. Introduction

Highly fluorescent materials with changeable optical properties have attracted considerable attention because of their valuable applications in chemistry, materials science, and biology [1]. Thus, the development of suitable fluorescent materials is essential for high-technology applications, such as organic optoelectronic devices (as OLEDs) [2], organic photovoltaic devices (OPVs) [3], organic field effect transistors [4], data storage devices [5], and sensors [6]. However, high fluorescent materials with bioimaging features are an extra advantage for practical applications in biomedical science [7,8,9]. In 2001, Tang and his co-worker reported that some fluorescent materials become highly fluorescent in their aggregate state, called aggregation-induced emission (AIE) [10], which is a totally opposite phenomenon of the conventional aggregation-caused quenching (ACQ) effect. Most fluorescent materials suffer for their non-emissive nature in their aggregate or condensed state (ACQ effect) due to strong intermolecular π–π interaction, which is harmful for their practical applications [11]. The great discovery of AIE phenomenon by Prof. Tang, made it easier for the synthesis of high luminescent materials with various biological applications [12]. Many fluorescent molecules show an AIE effect, but the most common feature of the AIE active molecules is that they have multi-aromatic systems with free rotating parts [13,14]. These AIE active molecules are classified into two categories: (i) AIE [15] where non-emissive materials become emissive in condense state and (ii) aggregation-induced emission enhancement (AIEE) where weakly emissive molecules show an emission enhancement on aggregation [16]. After discovery of the AIE phenomenon, it became one of the most popular scientific topics up to now and its study is growing very fast in various fields. Up to now, several AIE or AIEE active molecules have been reported and the corresponding possible applications and switching mechanisms properties have also been proposed [17]. A detailed explanation of the AIE phenomenon still needs to be considered, however, several explanations are reported for the support of the AIE mechanism, which are (i) restricted intramolecular rotation (RIR), (ii) reduced intermolecular π–π interactions, (iii) twisted intramolecular charge transfer (TICT), and photoinduced electron transfer (PET) [18].

Nowadays, AIE and AIEE active materials have been extensively used for their potential applications in biomedical science [19], in ions and explosive compounds detection [20,21], as an optoelectronic material [22], in the detection of active biomolecules [23], or in the detection of hazardous materials [24,25]. The fluorescent properties of some fluorophores are quenched because of intramolecular charge transfer (ICT) or the PET effect and become highly fluorescent on suppression of these effects in their aggregation state [26,27]. Among various AIE and AIEE active fluorophores, the ICT- or TICT-based fluorophores have attracted considerable attention due to their intrinsic electroactivity and photoactivity, which are beneficial for applications in advanced technology [28]. Thus, the use of ICT and AIE characteristics probes may have better advantages than using single characteristic AIE or ICT probes in the optoelectronic or biomedical field. However, it is difficult to synthesize the fluorophores with strong AIE and ICT characteristics because these two phenomena behave differently. Recently, Prof. Tang and his co-worker reported that a weak ICT probe showed a strong AIE phenomenon and a strong ICT probe showed a weak AIE phenomenon in the aggregation state [29]. Therefore, the development of strong ICT as well as strong AIE characteristics probes are still required for optoelectronics and biomedical applications. Some examples are reported based on the AIE and ICT mechanism, but their synthetic procedure is highly complicated [30].

**Figure 1:**
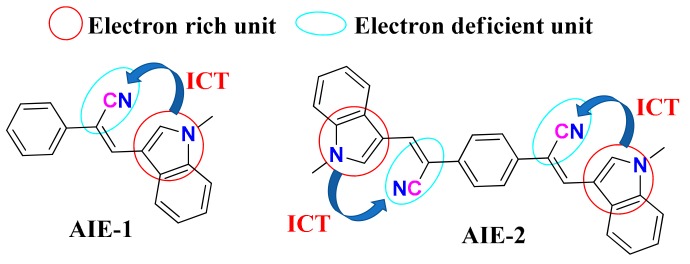
Structure of AIE-1 and AIE-2. ICT is abbreviated for intramolecular charge transfer (ICT).

On the other hand, cyano-functionalized fluorophores are well known for their intrinsic luminescent properties, highly photostability, mechanofluorochromism, and AIE phenomenon [31]. Recently, Prof. Tang reported that two cyano-functionalized derivatives showed distinct photophysical properties in their condensed state and two photon bioimaging applications [32]. Also, a red shift of emission from visible to the far red/near-infrared (NIR) region of cyano-functionalized derivatives were observed on aggregation by introducing donor-acceptor units in the molecular structure [33]. It was also reported that the AIE characteristics of cyano-functionalized derivatives were highly dependent on geometry [34]. Keeping in mind all the reported examples of cyano-functionalized derivatives, we were interested in developing some new donor-acceptor-containing cyano-functionalized derivatives with an easy synthesis procedure and from low-cost materials because of their high photostability, biomedical applications, and intrinsic luminescent properties. To fulfill the donor-acceptor requirement, a cyano-functional group was chosen as the electron acceptor as well as for the AIE core unit and an indole moiety was chosen for its strong electron donating ability. Indole derivatives with different *N*-alkyl chain length-dependent piezofluorochromic and AIE properties are well established [35]. Also, indole-containing transition metal complexes were reported for their highly selective human serum albumin (HSA) protein binding and controllable luminescent properties [36]. Thus, our major interest was to investigate the structural impact of the synthesized cyano-functionalized derivatives on the AIE or ICT effect and toward cytotoxicity and cell imaging studies. Therefore, we synthesized and characterized two cyano-functionalized fluorophores, AIE-1 and AIE-2, based on AIE/ACQ and ICT characteristics to investigate the structural-dependent aggregation behaviors, solvatochromism, cell cytotoxicity, and cell imaging applications (Figure 1). In AIE-1 and AIE-2, the indole moiety acted as an electron donor and the cyano-functional group acted as an electron acceptor unit. So, the electron-rich indole moiety transferred electrons to the electron-deficient cyano-functionalized phenyl ring through conjugated π-systems.

## 2. Results and Discussion

The two probes, AIE-1 and AIE-2, were synthesized by following an earlier reported procedure [37]. In brief, a Knoevenagel condensation reaction between 1-methylindole-3-carboxyaldehyde and phenylacetonitrile or 1,4-phenylenediacetonitrile yielded AIE-1 and AIE-2, respectively, in the presence of a base. The two probes, AIE-1 and AIE-2, were characterized by NMR and mass spectroscopy. These two probes were synthesized to see the structural impact on the aggregation, solvatochromism, cell toxicity, and cell imaging behaviors.

### 2.1. Electronic Feature of AIE-1 and AIE-2 by Density Functional Theory (DFT) Calculation

To see the detailed electronic features of AIE-1 and AIE-2, density functional theory (DFT) calculation was performed to optimize the structure and also for highest occupied molecular orbital (HOMO) and lowest unoccupied molecular orbital (LUMO) energy calculations. The optimized structures of these two probes are given in Figure S1**,** which shows a slightly twisted conformation. The single bond twisting is mainly found in the conjugated π-systems between the electron donor and acceptor unit of the probes.

**Figure 2:**
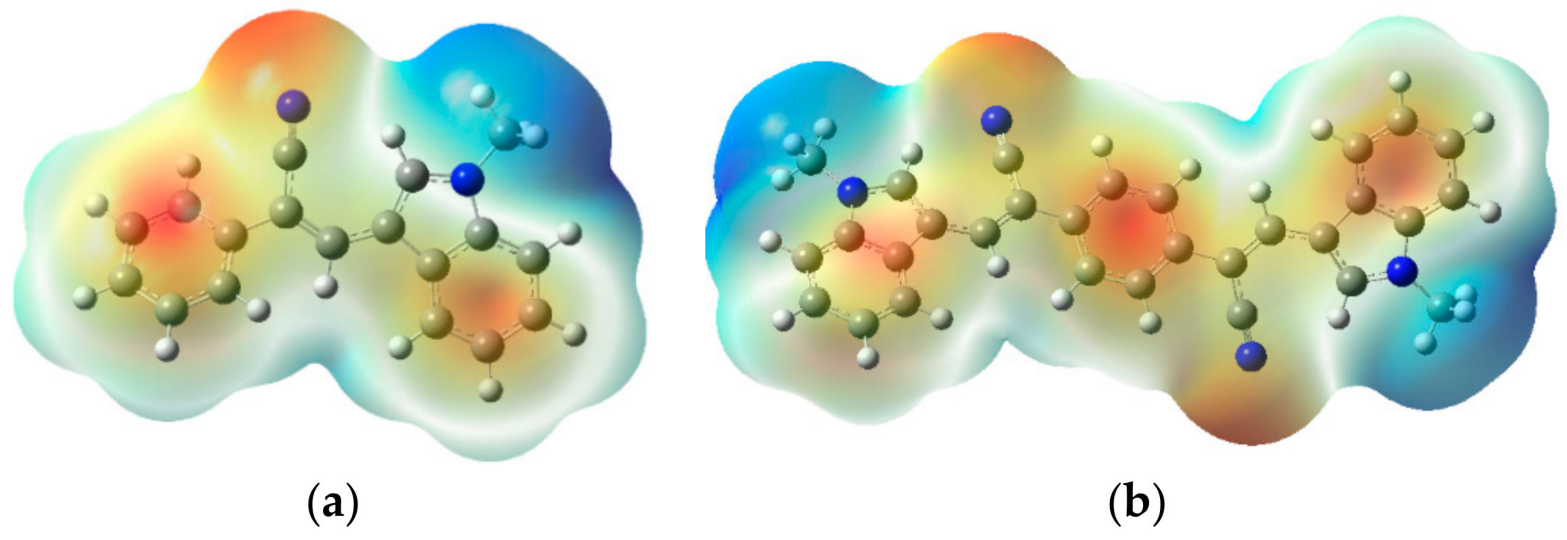
Molecular electronic potential diagrams of (**a**) AIE-1 and (**b**) AIE-2. Calculated by density functional theory (DFT) using B3LYP/6-31G as the basis set.

The HOMO of AIE-1 and AIE-2 were spread over whole molecules; major orbital loops are found in the indole unit. However, the LUMO were mainly found in the cyano-functionalized phenyl ring, but some orbital portions were in the indole unit (Figure S2). So, it was difficult to tell if the electron transfer takes place from the electron donor indole unit to the electron deficient cyano-functionalized phenyl ring. Thus, the molecular electronic potential (MEP) diagrams of AIE-1 and AIE-2 were obtained from a Gaussian to see the electronic distribution in the molecular surfaces. In Figure 2, the blue and red color surfaces refer to the electron-rich and electron-deficient units in the molecule [38]. As seen in Figure 2, the blue color surfaces were mainly found in the indole unit, which is the electron-rich center, and the red color surfaces were found in the rest of the molecule. Therefore, from the MEP diagrams of AIE-1 and AIE-2, we could easily tell that the electron transfer took place from the indole to the cyano-functionalized phenyl ring. The calculated HOMO and LUMO energy gaps of AIE-1 and AIE-2 are 3.612 and 3.083 eV, respectively (Table S1). The AIE-2 probe had a lower HOMO–LUMO energy gap than the AIE-1 because of higher π-conjugated systems present in AIE-2.

### 2.2. Aggregation Study by UV-Vis and Fluorescence Spectroscopy

**Figure 3:**
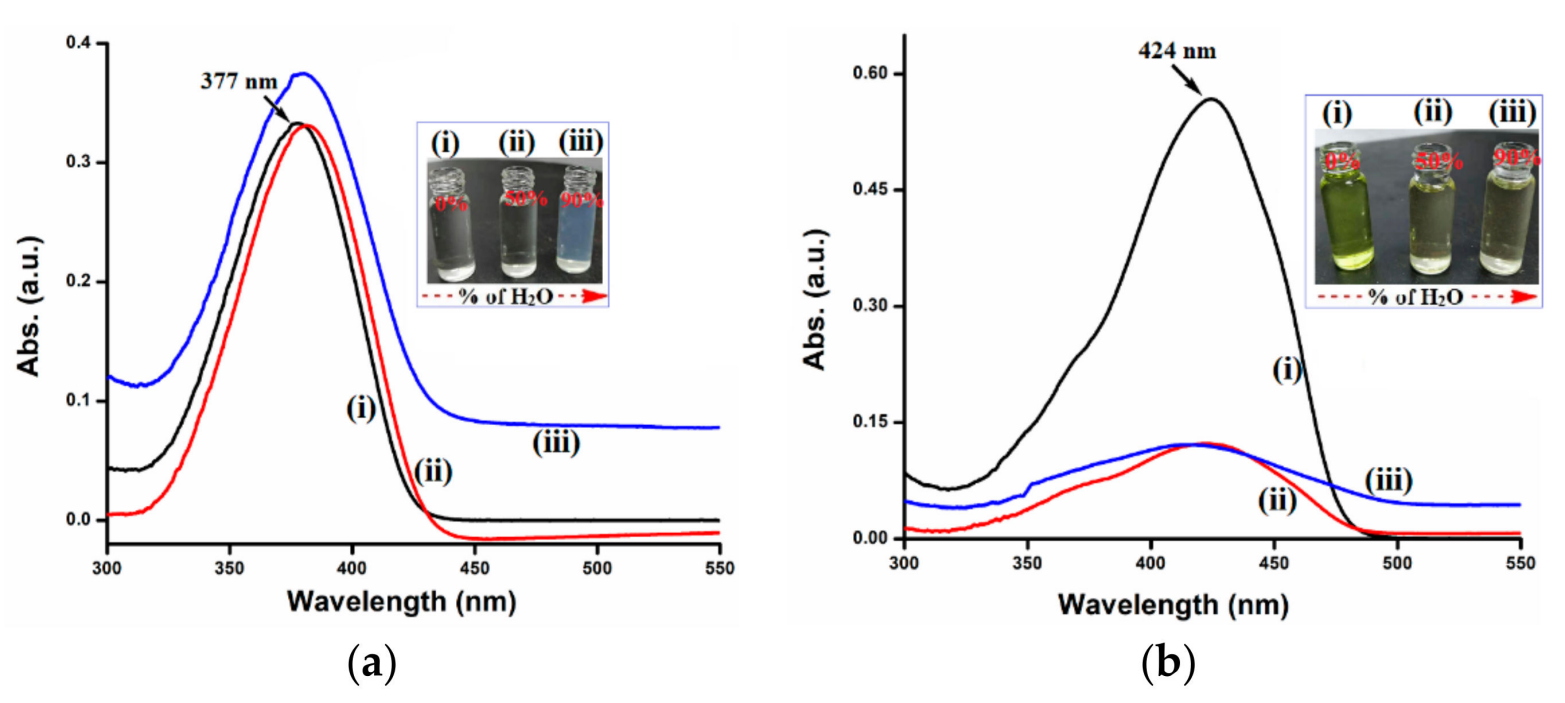
UV-Vis spectra of (**a**) AIE-1 (20 µM) and (**b**) AIE-2 (20 µM) in THF with (i) 0%, (ii) 50%, and (iii) 90% water. Inset figures show the color changes with different water fractions.

The HOMO–LUMO energy differences of AIE-1 and AIE-2 were reflected in their corresponding UV-Vis absorption band in THF solution. The UV-Vis absorption bands of AIE-1 and AIE-2 were found in at 377 nm and 424 nm, respectively, in THF solution (Figure 3). These absorption bands arose from the charge transfer transition from the donor indole to the acceptor cyano-functionalized phenyl unit of the probes. The AIE-2 probe absorbed at a higher wavelength than the AIE-1 probe because of more π-conjugated systems present in AIE-2 with a low HOMO–LUMO energy gap. More π-conjugated systems present in AIE-2 was also reflected in the corresponding color of the powder samples. The color of AIE-2 is yellowish-orange while AIE-1 is a yellow color with fewer π-conjugated systems (Figure S3).

The UV-Vis absorption spectroscopic studies of AIE-1 and AIE-2 in different THF-H_2_O (*v/v*) solvent mixtures revealed that both the probes form aggregated molecules in high water fractions. Probe AIE-2 showed huge absorption changes with the 50% water fraction, but no noticeable changes in the absorption value was observed for AIE-1 (Figure 3). Further increasing the water fraction to 90%, the absorption maxima of AIE-1 become uplifted baseline with slight increased absorption value. A similar increased baseline was observed for AIE-2 with the 90% water fraction, but the absorption value remained the same as that for the 50% water fraction. On increasing the water fraction, no noticeable changes in absorption maxima was observed, which indicates that both of the probes are stable in highly aqueous environments. However, the decreasing absorbance value is due to differences in solubility of the fluorophore in the THF-H_2_O solvent mixtures than in pure THF owing to the change in the dielectric constant of the solution [39,40]. The different patterns of spectral changes of AIE-1 and AIE-2 mainly depend on the molecular structure and possible formation of aggregates with decreasing solubility in high water fractions. The inset of Figure 3 shows the corresponding color changes with different water fractions of the two probes AIE-1 and AIE-2. 

**Figure 4:**
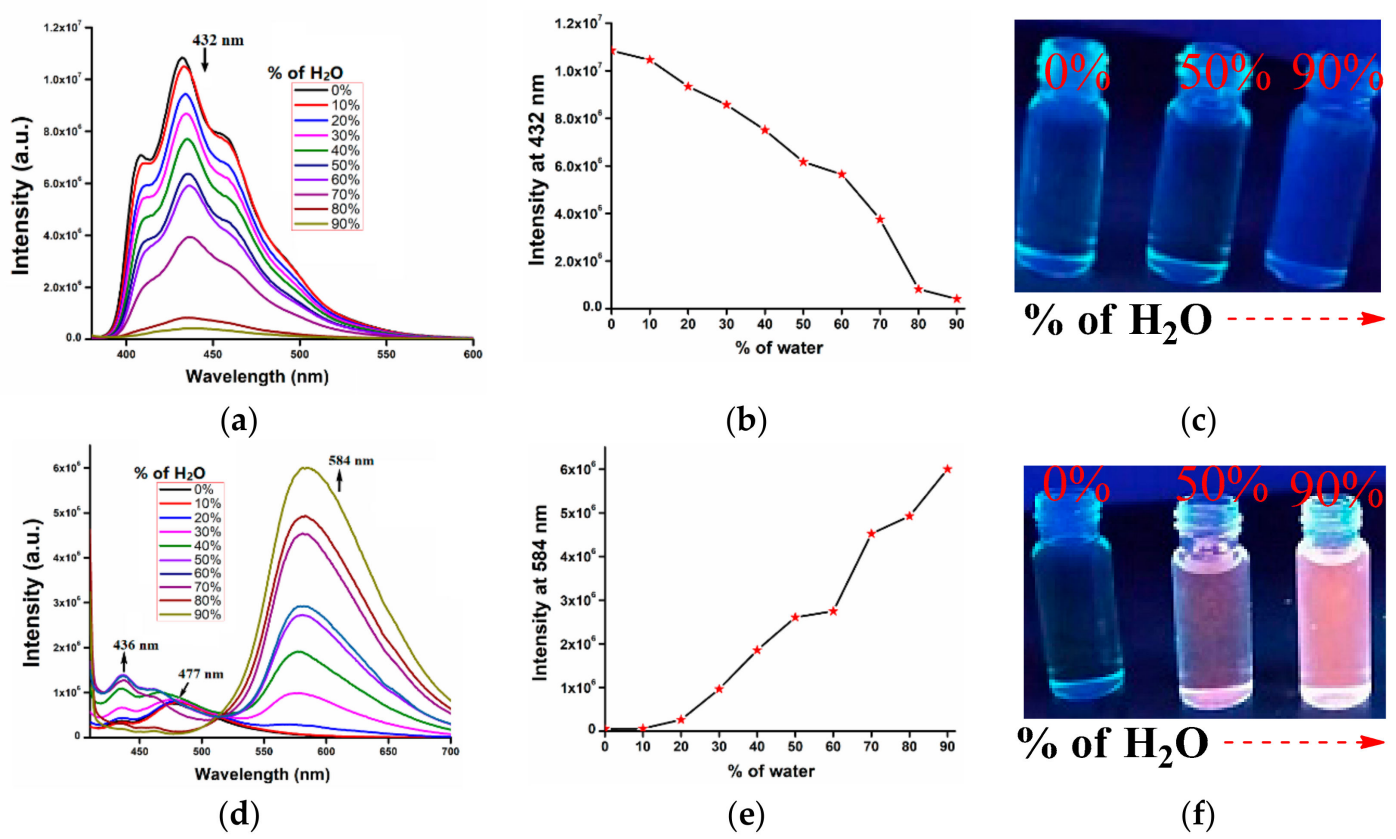
Fluorescence spectra of (**a**) AIE-1 (20 µM) excited at 365 nm and (**d**) AIE-2 (20 µM) excited at 400 nm in THF with (**b**) and (**e**) different water fractions. (**c)** and( **f**) Intensity vs. water fraction plots of AIE-1 and AIE-2. Fluorescence images of AIE-1 (20 µM) and AIE-2 (20 µM) under 365 nm UV lamp in THF with 0%, 50%, and 90% water.

To gain a better understanding of aggregation in solution, the emission spectra of AIE-1 and AIE-2 were recorded in different THF-H_2_O (*v/v*) solvent mixtures. The emission intensity at 432 nm of AIE-1 gradually decreased with increasing water fractions and became almost non-emissive with 80–90% water fractions (Figure 4a). Whereas, for **AIE-2**, the emission spectra at 477 nm gradually shifted to 584 nm with increasing water fractions and became highly emissive at 90% water (Figure 4d). Consequently, a new emission band at 436 nm of **AIE-2** with 20–70% water fractions was observed. Further increasing the water fractions from 80–90%, this emission band at 436 nm almost disappeared. Overall, the weakly emissive **AIE-2** probe became highly emissive on aggregation with high water fractions, showing AIEE. Similar types of AIEE behaviors were reported earlier in a THF-water solvent mixture of cyano-functionalized probes [41]. Not only the cyano-functionalized probes, but other functional groups containing probes have also been reported to have good AIEE active properties in different solvent mixtures, such as carbazole [42], diphenylamine [43], tetraphenylethene [44], hexaphenylsilole [45], and naphthalimide [46]. On the other hand, cyano-stilbene-based probes are also well known for their good mechanofluorochromic [47], thermochromic [48], and piezochromic [49] behaviors in their condensed state.

As both the probes, AIE-1 and AIE-2, contain donor and acceptor units in their molecular structure, the emission enhancement of AIE-2 can be attributed to the formation of aggregates with a stabilized ICT effect in high water fractions [50]. Also, the optimized structure of AIE-2 is in a slightly twisted conformation because of single bond rotation, but, on aggregation in aqueous medium, the twisted conformation becomes planar with no free single bond rotation (Figure S4b) [51]. As a result, the weakly fluorescent probe AIE-2 shows the AIEE property in a high water fraction in the aggregation state. It is a well-known mechanism that, on restriction of intramolecular motion, the non-emissive fluorophores become highly emissive on aggregation [52]. It was also reported that the weakly emissive cyano-stilbene derivatives become highly emissive with the conformational changes from twisted to planar on aggregation [53]. On the other hand, the AIE-1 probe shows quenching of emission on aggregation in different water fractions. The ACQ behavior of AIE-1 is due to conformational changes from twisted to planar with head to tail donor-acceptor arrangement through π–π interaction (Figure S4a). Most of the fluorophores show ACQ behavior on aggregation or in their condensed state because of strong π–π interaction [54].

The different aggregation behaviors of AIE-1 and AIE-2 were also reflected in their corresponding intensity vs. water fraction plots (Figure 4b,e). These two graphs clearly show that on gradually increasing the water fractions in THF solution of probes, the fluorescence intensity at 432 nm of AIE-1 gradually decreased and the intensity at 584 nm of AIE-2 gradually increased. The fluorescence images of AIE-1 and AIE-2 in THF and with different water fractions under 365 nm UV lamp are given (Figure 4c,f) to see the fluorescence changes with the naked eye. For AIE-1, the fluorescence changes in different water fractions are difficult to discriminant with the naked eye, whereas, for AIE-2, clear fluorescence changes are found with 50% and 90% water fractions from colorless to orange color emissions.

To understand the exact conformation or conformational changes in solution, the 2D-NOESY NMR of the AIE-1 probe was recorded and given as a representative case in Figure S13. As seen in Figure S13, we found that the *N*-methyl proton shows strong nuclear overhauser effect (NOE) coupling with protons Ha and He. Similarly, other weak NOE coupling was found between the protons Hb and Hd, Hb and Hc, Hc and Hi, and Hh and He. There was no NOE coupling between the Hd or Hb protons and indole ring protons. Thus, this result suggests that the indole moiety and the phenyl ring of the AIE-1 probe exist in the trans-conformation (inset structure of Figure S13). On the other hand, it is really difficult to tell the exact conformation of the probes after aggregation. However, it was reported earlier that, due to conformational changes from twisted to planer, the weakly emissive cyano-stilbene derivatives become highly emissive on aggregation [53].

### 2.3. Aggregation Confirmed by Scanning Electron Microscope (SEM) and Dynamic Light Scattering (DLS) Studies

**Figure 5:**
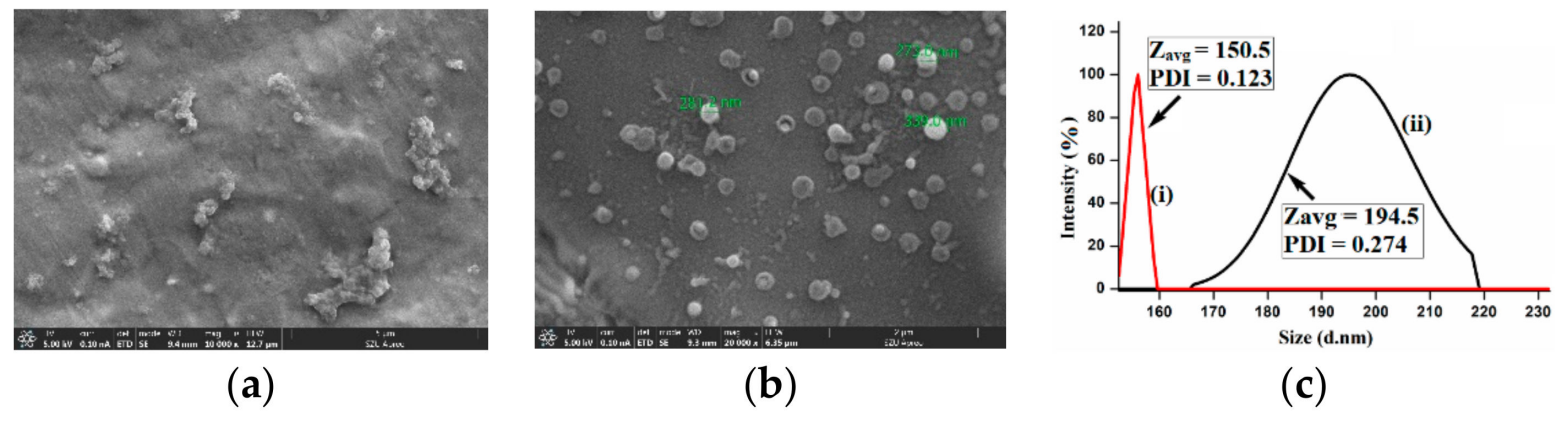
SEM images of (**a**) AIE-1 and (**b**) AIE-2 taken from drop-cast probes in THF-H_2_O (1:9, *v/v*). (**c**) DLS-based particle size analysis of AIE-1 in (i) 100% THF and (ii) THF-H_2_O (1:9, *v/v*) solvent mixtures.

From UV-Vis and fluorescence spectroscopic studies, we found that AIE-1 and AIE-2 show aggregation behaviors in THF-H_2_O solvent mixtures. But, it was difficult to tell the exact types of aggregation in higher water fraction from these two experiments. Thus, scanning electron microscope (SEM) and dynamic light scattering (DLS) experiments were carried out to investigate the morphology and average particle size of AIE-1 and AIE-2 on aggregation in higher water fraction. SEM images of AIE-1 and AIE-2 in THF and THF-H_2_O solvent mixtures are given in Figure 5a,b and Figure S5. From the SEM studies, we found that both the probes form nano particles in 100% THF solutions and the average particle size is in the range from 140 to 200 nm for AIE-1 and from 200 to 260 nm for AIE-2. Bigger structures form bigger particles in non-aggregated solution. But, on aggregation with 90% water, both the probes formed different morphologies. Probe AIE-2 formed bigger nano-particles (280–340 nm) on aggregation compared to the particle size of the non-aggregated probe. Whereas for probe AIE-1, coagulated-type of morphology was observed on aggregation with 90% water (Figure 5a).

This type of morphology is formed by aggregates of small particles associated with solvent molecules. It is known that, on aggregation, the particle size of the fluorophore is increased [55]. Also, a similar type of Field Emission Scanning Electron Microscopy (FESEM) morphology was reported on aggregation of cyano-stilbene derivatives [37]. Furthermore, DLS experiments of AIE-1 and AIE-2 were carried out to confirm the aggregation phenomenon in THF-H_2_O solvent mixtures. The average particle size of AIE-1 and AIE-2 increased when increasing the water fractions from 0–90% in THF solvent (Figure 5c and Figure S6). The average particle size of AIE-1 in THF solvent was 150 nm (Figure 5c), but the particle size of AIE-1 increased to 194 nm on aggregation. A similar result was found in the case of AIE-2, where the particle size of AIE-2 increased with increasing the water fraction (Figure S6). The increasing particle size is due to the accumulation of particles associated with solvent molecules to form larger aggregates of probes in higher water fractions. Differences in the particle size of AIE-2 or AIE-2 were observed in SEM and DLS because of solvent-assisted particle formation in the DLS experiment.

Thus, the SEM and DLS results suggest that the AIE-1 or AIE-2 probe forms aggregates in higher water fractions and, also, these two experimental results are in good agreement with spectroscopic studies in THF and THF-H_2_O solvent mixtures.

### 2.4. Emission of AIE-1 and AIE-2 in Different Solvents and in Different Concentrations:

**Figure 6:**
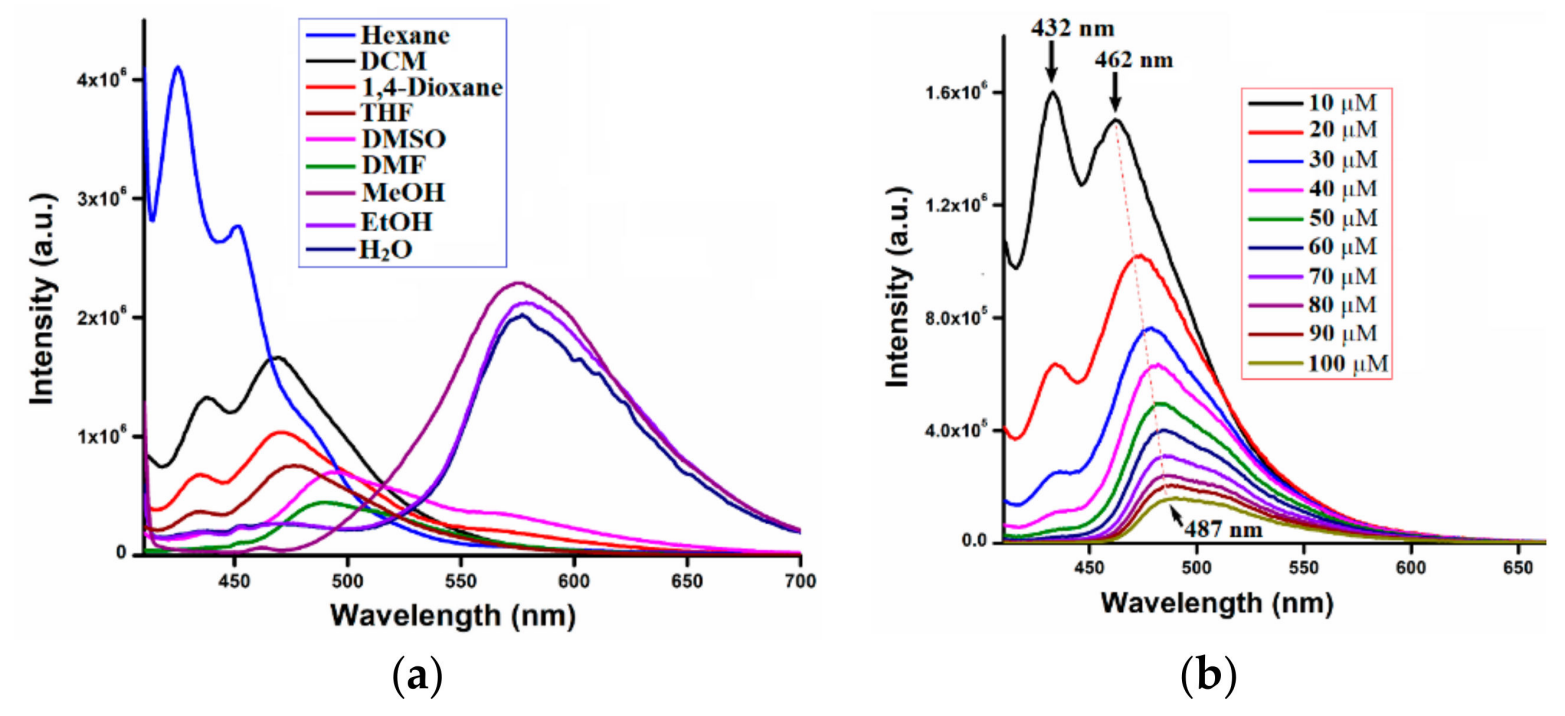

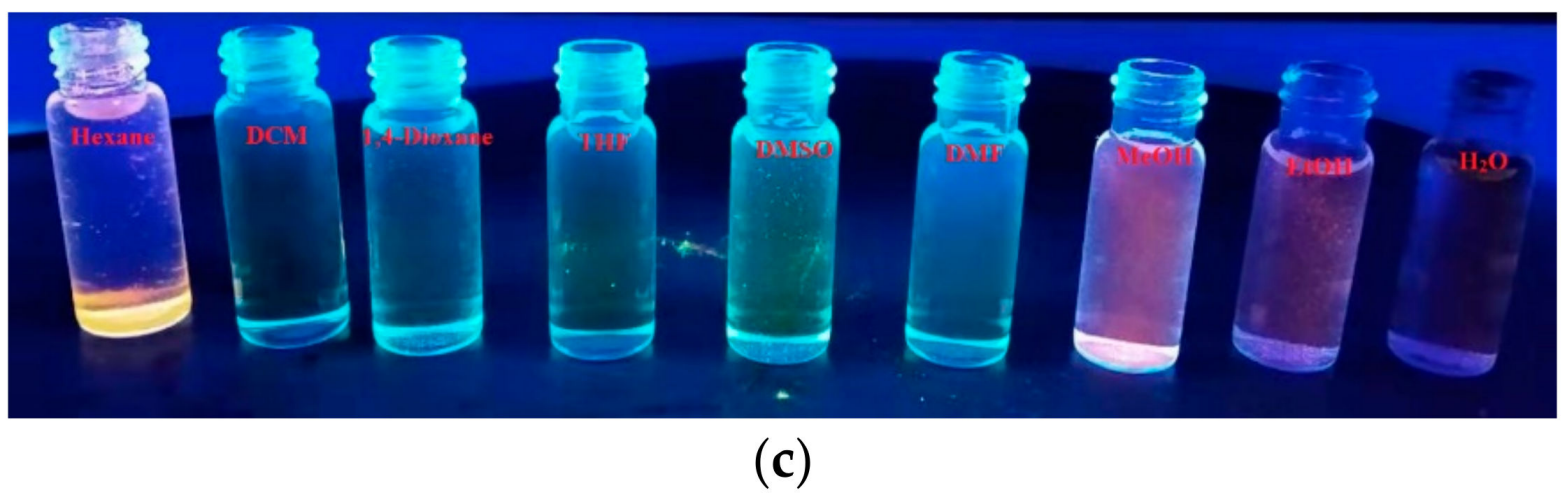
Fluorescence spectra of AIE-2 (20 µM) in different (**a**) solvents and (**b**) concentrations excited at 400 nm and (**c**) fluorescence images of AIE-2 (20 µM) in different solvents under 365 nm UV light.

The DFT-optimized structures of AIE-1 and AIE-2 showed that both the probes exist in a twisted conformation (Figure S1), which is favorable for active intramolecular single bond rotations of donor or acceptor moieties in solutions. Therefore, it is possible for the two AIE-1 and AIE-2 to show the solvent-dependent or concentration-dependent emission behaviors in solution. To investigate the solvatochromism behaviors of AIE-1 and AIE-2, the emission spectra of these probes were recorded in different solvents of varying polarities. As a representative example, the emission spectra of AIE-2 in different solvents and in different concentrations are given in Figure 6a,b and the corresponding fluorescence images in different solvents of AIE-2 under 365 nm UV light are given in Figure 6c. The AIE-2 probe showed two emission bands at 424 nm and 451 nm in nonpolar solvent hexane, but on increasing the solvent polarities from low to moderate (e.g., DCM, THF, and 1,4-dioxane), the fluorescence intensity gradually decreased and a red shift of emission was observed. At the same time, the more prominent emission peak at 424 nm in hexane became less prominent, while the less prominent emission peak at 451 nm became more prominent with 20–25 nm red shift on increasing the solvent polarities. Further increasing the solvent polarities to high polar solvents (e.g., DMF and DMSO), the emission peak of AIE-2 further shifted to 488 nm (in DMF) and 494 nm (in DMSO) with a single prominent peak. It is known that on increasing the solvent polarities from low to high, the emission peak of a donor-acceptor moiety containing fluorophores shows a bathochromic shift with a decrease in the fluorescence intensity and quantum efficiency [56]. Thus, the change of emission of AIE-2 on increasing the solvent polarities was not unusual. On increasing the solvent polarities from a non-polar to a high polar solvent, the excited state of ICT-based probes was more stabilized in polar solvents compared with non-polar solvents. As a result, the excitation energy becomes lower in polar solvents and, hence, a remarkable bathochromic shift of emission was observed [57].

Interestingly, we observed the emission red shift around 80–90 nm when the AIE-2 probe was dissolved in protic solvents, such as methanol, ethanol, and water. In protic solvents, the AIE-2 probe showed emission peaks from 575–584 nm on excitation at 400 nm. In the aggregation study portion, we already discussed the aggregation behavior of AIE-2 in THF-H_2_O solvent mixtures and mentioned that the aggregated AIE-2 probe emitted at 584 nm. So, it is obvious that in 100% water the AIE-2 probe will show aggregation emission at 584 nm with low intensity. The red shift of emission in protic solvents is because of strong hydrogen bonding interactions between AIE-2 probe and solvent molecules in the excitation state [58] and also, because of less solubility, the probe become aggregated. In the case of AIE-1, the changes in emission when increasing the solvent polarity from low to high polar were different. On increasing the solvents polarity, the emission intensity of **AIE-1** gradually decreased and became almost non-emissive in protic solvent (Figure S7). No noticeable changes in emission wavelength were observed on increasing the solvent polarities. The different solvatochromism effects of AIE-1 and AIE-2 suggest that the AIE-2 probe has a larger dipolar moment in the excited state than the ground state, which is due to more charge redistribution in the molecular structure of the AIE-2 probe compared with the AIE-1 probe [59].

Furthermore, the emission spectra of AIE-1 and AIE-2 were recorded in different concentrations to see the aggregation behavior in different concentrations (Figure 6b and Figure S8). The emission intensity of AIE-2 gradually decreased with a slight red shift in emission wavelength on increasing the concentrations of the probes. No such shift in emission wavelength was observed for AIE-1 on increasing the probe concentrations. On increasing the probe concentration, each single AIE-1 or AIE-2 probe came closer and formed aggregated AIE-1 or AIE-2 probes in the high concentration solution. Therefore, the intramolecular motions of the probes are blocked in the higher concentration solutions and, as a result, lower emission intensity with a slight red shift in emissions of the probes were observed.

On the other hand, it is difficult to identify the fluorescence changes of AIE-2 on increasing the solvent polarities from hexane to DMSO through the naked eye under day light (Figure S9) or under 365 nm UV light (Figure 6c). However, the fluorescence changes were clearly visualized when changing the aprotic solvent to the protic solvent. The AIE-2 probe showed an intense orange color in protic solvents, but almost no color in aprotic solvents under 365 nm UV lamp (Figure 6c). However, the yellow color solution of AIE-2 in aprotic solvents become colorless in protic solvents (Figure S9). No noticeable color or emission changes were observed for AIE-1 under day light or under 365 nm UV light (Figure S10).

### 2.5. Cytotoxicity and Cell Imaging Applications of AIE-1 and AIE-2:

**Figure 7:**
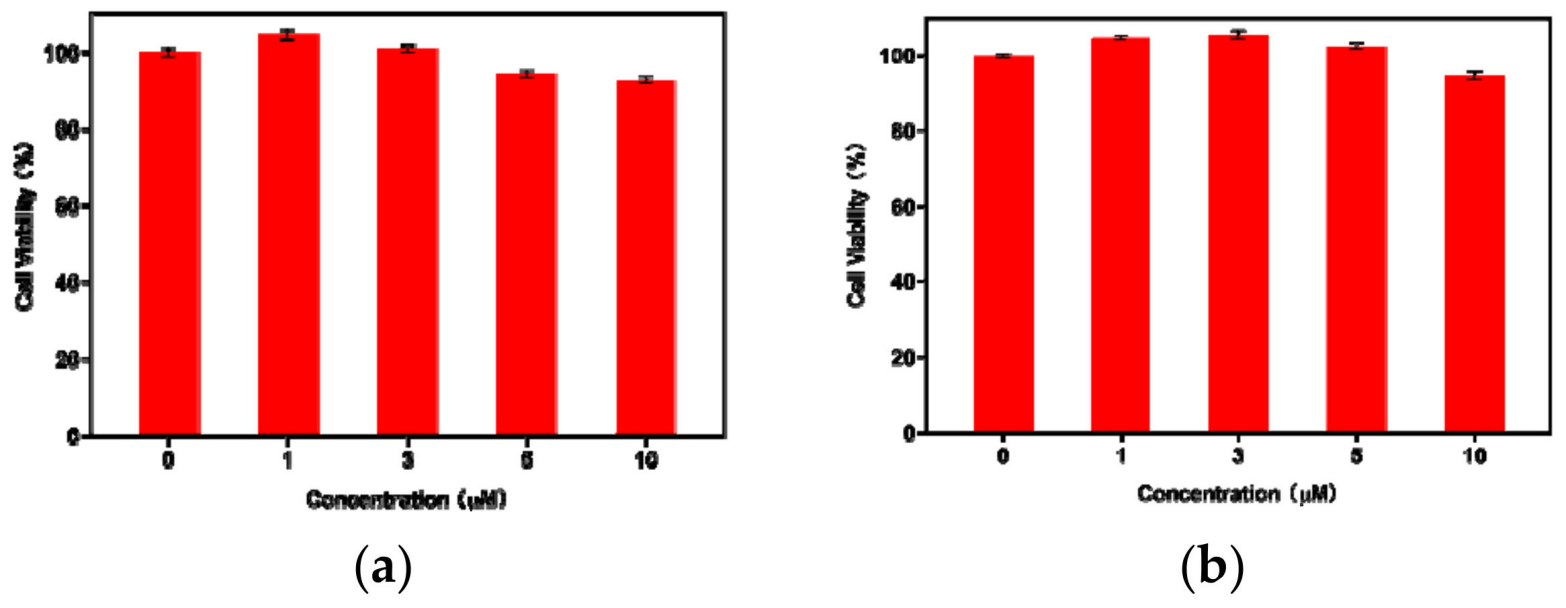
Cytotoxicity results of (**a**) AIE-1 and (**b**) AIE-2 on HeLa cells using concentrations from 0–10 µM with 24 h incubation time.

Cell imaging applications by AIE active probes are well known in the field of biochemistry [60]. In most of the cases, HeLa cells are used as a cell model for cell imaging studies with AIE active probes [61]. Therefore, we explored the aggregated AIE-1 and AIE-2 probes for imaging of living cells using HeLa cells. Before using the probes for cell imaging, it was crucial to investigate the cytotoxicity of AIE-1 and AIE-2 in living cells using a cell counting kit-8 (CCK8) assay. The cell viability percentage vs. concentration plots of AIE-1 and AIE-2 are shown in Figure 7 and Figure S11 with different incubation times.

For the cell viability test, the concentrations of the AIE-1 and AIE-2 probes varied from 0–10 µM solution in phosphate buffered saline (PBS) (with <0.2% DMSO). The cell viability results of AIE-1 and AIE-2, as shown in Figure 7, demonstrate that the HeLa cells exhibited good viability (more than 90%), even after 24 or 48 h of incubation. This result also indicates that the AIE-1 and AIE-2 probes are biocompatible up to 10 µM solution for intracellular imaging of living cells and these probes also show low cytotoxicity to HeLa cells

**Figure 8:**
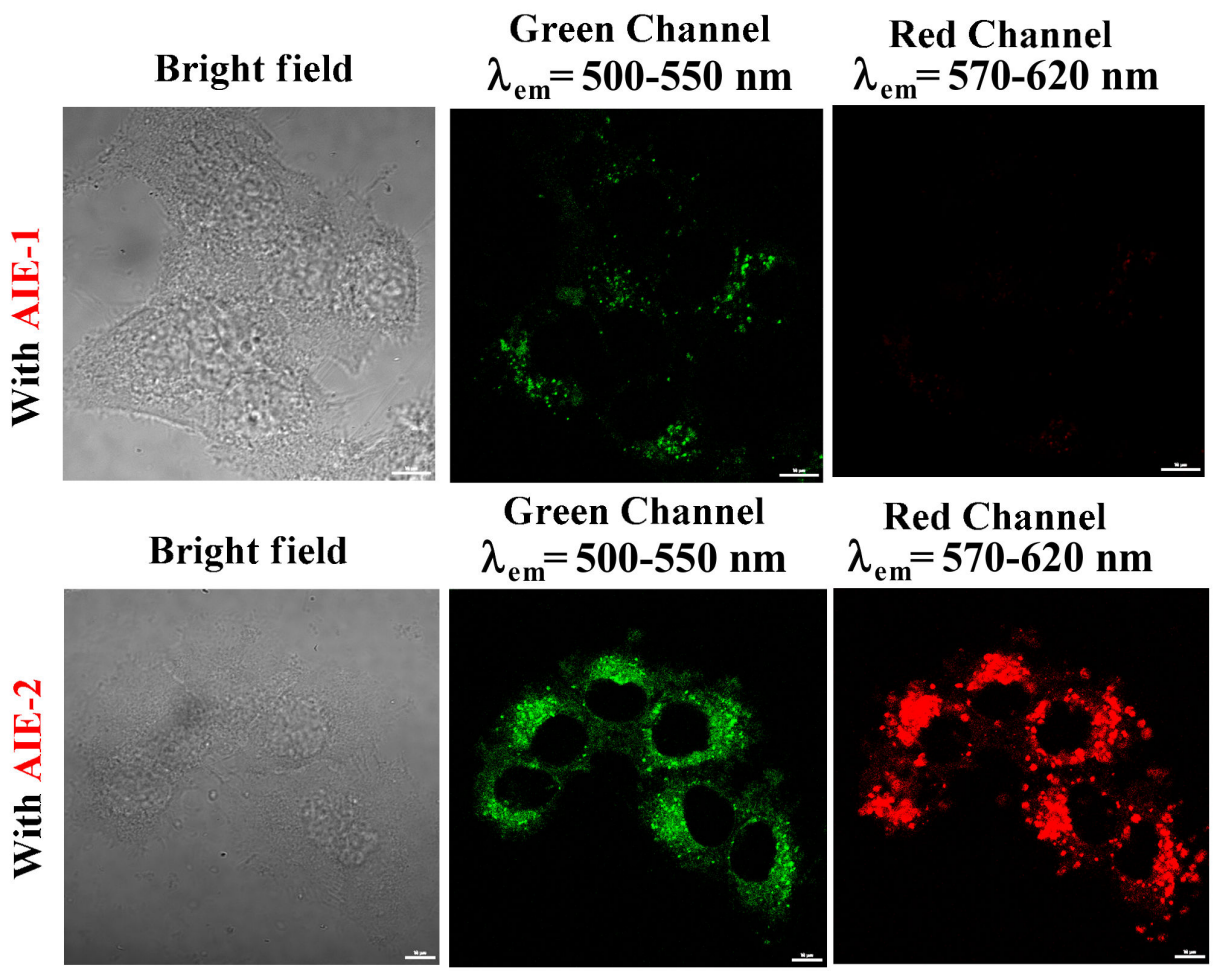
HeLa cell images in bright field, green channel (λ_em_ = 500–550 nm), and red channel (λ_em_ = 570–620 nm), incubated with 3 µM solution of AIE-1 and AIE-2. The scale bars for all the images are 10 μm.

For cell imaging studies, 3 µM solution of AIE-1 and AIE-2 were incubated for 24 h at 37 °C and then the cells were washed in PBS buffer to remove the extra probes. Fluorescence images of HeLa cells were collected by confocal laser scanning microscope using 405 nm laser light and the cell images in bright field and green (λ_em_ = 500–550 nm) and red (λ_em_ = 570–620 nm) channels are given in Figure 8. The bright field images, after treatment with probes AIE-1 and AIE-2, showed good cell viability and, with spindle morphology, suggests the high biocompatibility of both the AIE-1 and AIE-2 probes inside the cell.

Fluorescence images of HeLa cells with the AIE-1 probe display light green fluorescence and almost no red fluorescence. It was obvious that the aggregated probe AIE-1 will show weak green fluorescence inside the HeLa cell because probe AIE-1 forms weakly emissive aggregated probes inside the HeLa cell. However, when HeLa cells were incubated with probe AIE-2, green and red fluorescence images of HeLa cells were noted. The green and red fluorescence images of HeLa cells with probe AIE-2 were observed because of monomer and aggregated emissions of probe AIE-2 inside the HeLa cell. These results are consistent with the spectral changes of AIE-1 and AIE-2 in THF-H_2_O solvent mixtures (Figure 4). Thus, the aggregated AIE-1 and AIE-2 probes may be useful in the future for biomedical sensing purposes for in vivo applications.

## 3. Experimental Section

### 3.1. Details of General Instruments and Materials

All the starting materials and solvents for the synthesis of AIE-1 or AIE-2 and for other experimental purposes were purchased from commercial suppliers and used without further purification. The THF-H_2_O solvent mixtures for the AIE experiment with 0–90% water fractions were prepared by adding the exact amount of distilled water to the THF solvent with a fixed concentration of probes and volume of 20 µM and 2 mL, respectively. The UV-Vis and fluorescence spectra of AIE-1 and AIE-2 in different THF-H_2_O solvent mixtures were recorded on a Cintra 2020 UV-Vis and Horiba Fluoromax-4 spectrophotometer, respectively, using a 10 mm path length quartz cuvettes. For fluorescence spectroscopic studies, we used a 3 nm slit width and 2 mL solution for each experiment and recorded at room temperature with appropriate excitation wavelength. The ^1^H- and ^13^C-NMR spectra of AIE-1 and AIE-2 were recorded on a Bruker-500 MHz (Karlsruhe, Germany) spectrometer using DMSO-*d_6_* solvent with TMS (tetramethylsilane) as an internal standard. The electrospray ionization mass spectrometry (ESI-MS) of probes were obtained by using a mass quadrupole time-of-flight tandem mass spectrometer. The ground state optimized structures of AIE-1 and AIE-2 were optimized by density functional theory (DFT) using B3LYP/6-31G (d, p) level basis set with Gaussian 09W software. Other experimental details, such as cell toxicity, cell imaging, and stock solution preparation, are available as Supplementary Materials.

### 3.2. Synthesis and Characterization

**Scheme 1:**
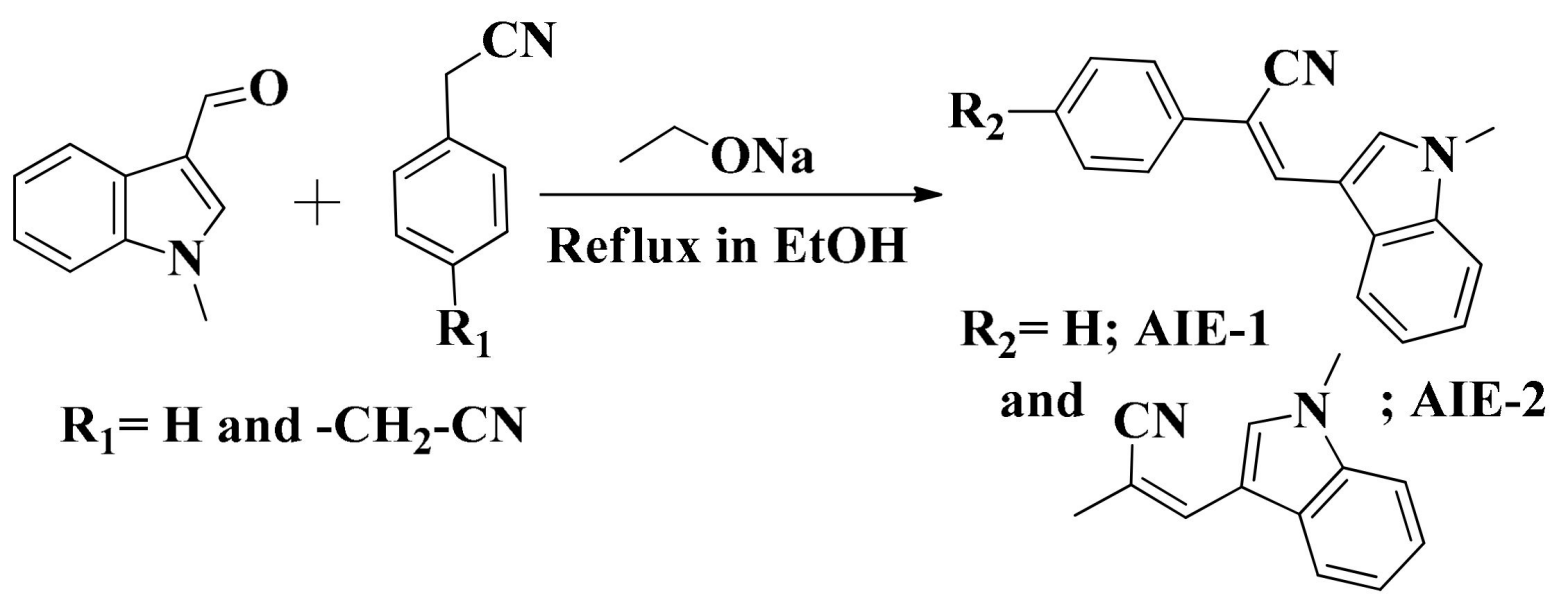
Synthetic strategy for synthesis of **AIE-1** and **AIE-2**.

The schematic diagram of the synthesis of AIE-1 and AIE-2 is given in Scheme 1 and followed the earlier reported procedure [37]. In brief, phenylacetonitrile (5 mmol, 1 eq.) and sodium ethoxide (5 mmol, 1 eq.) were dissolved in anhydrous ethanol (15 mL). After 10–15 min stirring at room temperature, 1-methylindole-3-carboxyaldehyde (5 mmol, 1eq.) was added to the reaction mixture and refluxed overnight at 70 °C. Upon cooling the reaction mixture, a yellow precipitate was obtained and the precipitate was filtered followed by several washes with fresh ethanol. The obtained precipitate was dried under vacuum for 2 h to afford yellow crystalline probe AIE-1. Whereas for the synthesis of AIE-2, we followed the same procedure as for AIE-1 but, instead of phenylacetonitrile, we used 0.5 eq. of 1,4-phenylenediacetonitrile to get yellowish-orange probe AIE-2.

*AIE-1.* Yellow crystalline solid, yield 80%. ^1^H-NMR (500 MHz, DMSO-*d_6_*): 8.36 (s, 1H), 8.23 (s, 1H), 8.11 (d, J = 10, 1H), 7.80 (d, J = 10, 2H), 7.58 (d, J = 10, 1H), 7.48 (t, J = 10, 2H), 7.36 (t, J = 10, 1H), 7.31 (t, J = 10, 1H), 7.24 (t, J = 10, 1H), 3.95 (s, 3H). ^13^C-NMR (125 MHz, DMSO-*d_6_*): 135.8, 133.8, 133.3, 130.1, 128.4, 127.2, 127.1, 124.3, 122.3, 120.3, 119.2, 118.3, 110.0, 109.3, 101.8, 32.7. ESI-MS (positive mode) calculated for C_18_H_14_N_2_ [M + H^+^]: 259.12; Found: 259.12.

*AIE-2.* Yellowish-orange solid, yield 85%. ^1^H-NMR (500 MHz, DMSO-*d_6_*): 8.40 (s, 2H), 8.32 (s, 2H), 8.16 (d, J = 10, 2H), 7.90 (s, 4H), 7.60 (d, J = 10, 2H), 7.33 (t, J = 10, 2H), 7.27 (t, J = 10, 2H), 3.97 (s, 6H). ^13^C-NMR (125 MHz, CDCl_3_): 136.5, 135.6, 134.6, 129.6, 128.8, 126.0, 123.3, 121.5, 120.2, 119.2, 118.2, 110.3, 105.9, 33.6. ESI-MS (positive mode) calculated for C_30_H_23_N_4_ [M + H^+^]: 439.19; Found: 439.19.

## 4. Conclusions

In conclusion, we achieved AIEE active probe AIE-2 by introducing more substitution in the molecular structure of the ACQ active probe AIE-1. The AIEE/ACQ behaviors were explained by the twisted to planer conformational changes, including the restriction of intramolecular single bond rotation in their aggregation state. This AIEE or ACQ results mainly depended on the types of stacking of AIE-1 or AIE-2 molecules in their aggregation state. The aggregation behaviors of AIE-1 and AIE-2 were studied by UV-Vis and fluorescence spectroscopy and were confirmed by SEM morphology and DLS-based particle size analysis studies. Furthermore, we investigated the role of different solvents and different concentrations of probes on their emissions and aggregation behaviors. Probe AIE-2 showed a bathochromic shift of emission in higher polar solvents and in higher probe concentrations. The DFT calculation study suggested that the indole moiety acts as an electron donor, the cyano-functionalized phenyl ring acts as electron acceptor, and the ICT process takes place from indole to cyano-functionalized phenyl ring through conjugated π-systems. On the other hand, both the probes showed less cytotoxicity on HeLa cells and probes AIE-1 and AIE-2 are suitable candidates for live cell imaging application. Thus, the overall outcome of this study was that the aggregated AIE-2 probe can be a good candidate for applications in optoelectronics or in biomedical science.

## Supplementary Materials

The following are available online at https://www.mdpi.com/1420-3049/25/3/585/s1, Figure S1: Optimized structures of (a) AIE-1 and (b) AIE-2. Structure optimized by DFT using B3LYP/6-31G as the basis set; Figure S2: (a,c) HOMO and (b,d) LUMO of AIE-1 and AIE-2, respectively; Figure S3: Probe (a) AIE-1 and (b) AIE-2 under (i) day light and (ii) 365 nm UV light; Figure S4: Different arrangements of (a) AIE-1 and (b) AIE-2 in THF and THF-H2O solvent mixture; Figure S5: SEM images of (a) AIE-1 and (b) AIE-2 in 100% THF by drop coast method; Figure S6: DLS-based particle size analysis of AIE-2 in (a) 100% THF and (b) THF-H2O (1:9, *v/v*); Figure S7: Fluorescence spectra of AIE-1 in different solvents; Figure S8: Fluorescence spectra of AIE-1 in different concentrations; Figure S9: Images of AIE-2 in different solvents under day light; Figure S10: Images of AIE-1 in different solvents under day and 365 nm UV light; Figure S11: Cytotoxicity results of (a) AIE-1 and (b) AIE-2 on HeLa cells using concentrations from 0–10 µM with 48 h incubation time; Figure S12: ^1^H-NMR (500 MHz, DMSO-*d6*) spectra of AIE-1; Figure S13: 2D-NOESY (500 MHz, DMSO-d6) spectrum of AIE-1; Figure S14: ^13^C-NMR (125 MHz, DMSO-*d6*) spectra of AIE-1; Figure S15: Mass spectra of AIE-1; Figure S16: ^1^H-NMR (500 MHz, DMSO-*d6*) spectra of AIE-2; Figure S17: ^13^C-NMR (125 MHz, CDCl_3_) spectra of AIE-2; Figure S18: Mass spectrum of AIE-2; Table S1: Details on the optimized energy and HOMO–LUMO energy gap of AIE-1 and AIE-2.
