# Supplementary material for: ICT and AIE Characteristics Two Cyano-Functionalized Probes and Their Photophysical Properties, DFT Calculations, Cytotoxicity, and Cell Imaging Applications
Source: Molecules. 2020 Jan 29;25(3):585. doi: 10.3390/molecules25030585 (PMC7037400; doi:10.3390/molecules25030585)
Supplement: Supplementary file 1 [file molecules-25-00585-s001.pdf]

Supporting Information

for

ICT and AIE Characteristics Two Cyano-Functionalized Probes and Their Photophysical Properties, DFT Calculations, Cytotoxicity and Cell Imaging Applications

Arup Tarai,[§] Meina Huang,[§] Pintu Das, Wenhui Pan, Jianguo Zhang, Zhenyu Gu, Wei Yan, Junle Qu* and Zhigang Yang*

Center for Biomedical Photonics, Key Laboratory of Optoelectronic Devices and Systems of Ministry of Education and Guangdong Province, College of Physics and Optoelectronic Engineering, Shenzhen University, Shenzhen 518060, China
Corresponding e-mail: jlqu@szu.edu.cn and zhgyang@szu.edu.cn

Preparation of stock solution: The stock solutions of probe **AIE-1** and **AIE-2** were prepared in DMSO of 1 mM concentration and kept in refrigerator for further usage. For spectroscopic studies we used 40 μL of 1 mM probe stock solution in each time and further diluted with respective solvents for respective studies to 2 mL probe solution.

For **SEM** images of drop (20 μM) cast sample of **AIE-1** and **AIE-2** on glass plates covered with Al-foil were taken by using SEM High Resolution Scanning Electron Microscope FEI APREO S from Netherlands.

For **DLS** experiment, the probe **AIE-1** or **AIE-2** solution in THF and THF-H_2O were prepared and equilibrated for 60 minutes before use the prepared sample for experiment. The DLS based particle sizes of **AIE-1** and **AIE-2** were measured by using NanoBrook 90Plus Zeta equipment.

Cytotoxicity Assay: Cytotoxicity assays were carried out using HeLa cells. Cell viability was determined using CCK8 assay. 10000 cells per well were seeded in a 96-well plate and incubated in a humidified incubator for adherence. After 24 h of culturing, the medium in each well was replaced by 100 μL of fresh medium (RPMI-1640) containing different concentrations (0, 1, 3, 5, 10 μM) of **AIE-1** and **AIE-2**, respectively. The solution was filtrated by 0.22 μm sterile filter. The volume fraction of DMSO was below 0.2%. After 24 h, CCK-8 reagent diluted by 1640 medium (10%) and was added to each well after the removal of culture media and incubated for 1 h. Following that, the absorbance was measured at 450 nm on a plate reader (Spectra Max M5, Molecular Devices, Sunnyvale, CA). Each trial was performed with six parallel wells. Cell viability rate was determined as VR = (As-Ab)/(Ac-Ab) × 100%, where As is the absorbance of the experimental group, Ac is the absorbance of the control group (no **AIE** probe), and Ab is the absorbance of

the blank group (no cells).

Cell imaging: Before imaging on a microscope, 1 mL HeLa cells at a certain density were seeded in the glass bottom culture dishes with cover (NEST). The cells were incubated in a humidified atmosphere containing 5% (v/v) CO_2 at 37 °C and allowed to adhere for 24 h. They were then subjected to the imaging experiments. For the cell imaging, the cells were incubated with 3 μM of the **AIE-1** or **AIE-2** in the culture medium for 24h, and then the medium was removed. The residual probe was removed by washing three times using phosphate buffered saline (PBS) before imaging. Finally, confocal fluorescence imaging was carried out by Nikon fluorescence microscope equipped with following excitation channels and corresponding emission collection range (λex= 405 nm, emission range: 500-550 nm (Green channel) and 570-620 nm (Red channel).

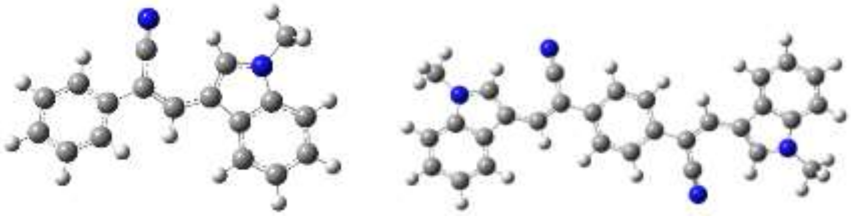

(a) (b)

Figure S1: Optimized structures of (a) **AIE-1** and (b) **AIE-2**. Structure optimized by DFT using B3LYP/6-31G as basis set.

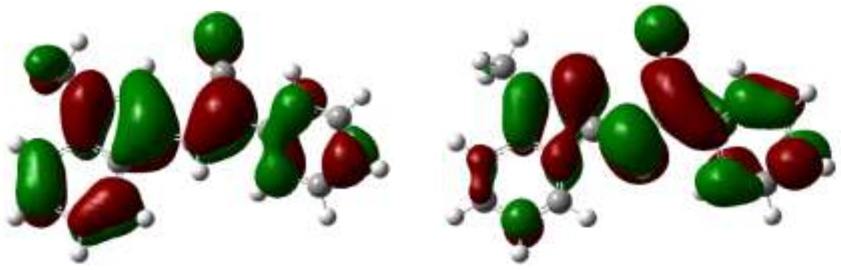

(a) (b)

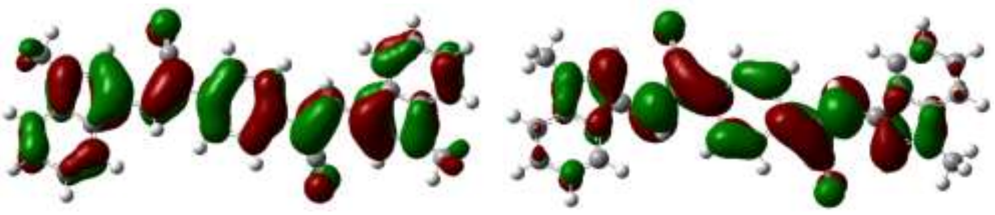

(c) (d)

Figure S2: (a, c) HOMO and (b and d) LUMO of **AIE-1** and **AIE-2** respectively.

Table S1: Details optimized energy and HUMO-LUMO energy gap of **AIE-1** and **AIE-2**.

Probe	HOMO (eV)	LUMO (eV)	Energy differences (HOMO-LUMO) in eV
**AIE-1**	- 5.6041	-1.9921	-3.612
**AIE-2**	- 5.3394	- 2.2560	-3.0834

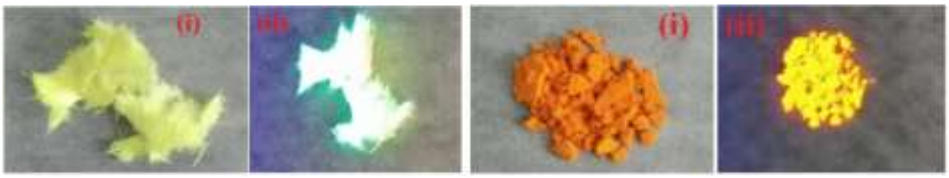

(a) (b)

Figure S3: Probe (a) **AIE-1** and (b) **AIE-2** under (i) day light and (ii) 365 nm UV light.

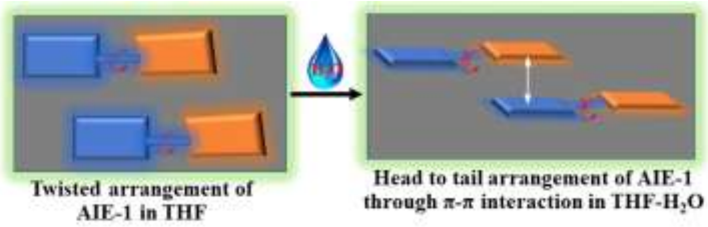

(a)

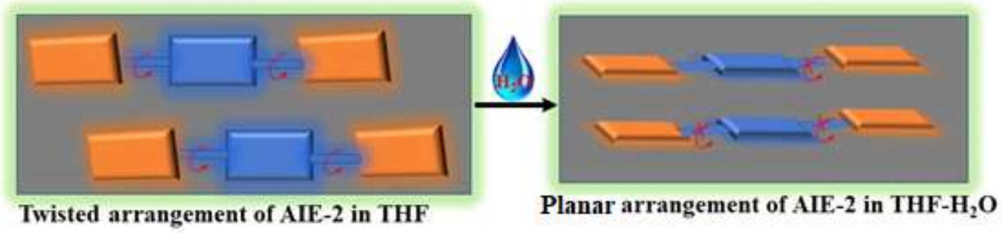

(b)

Figure S4: Different arrangements of (a) **AIE-1** and (b) **AIE-2** in THF and THF-H_2O solvent mixture.

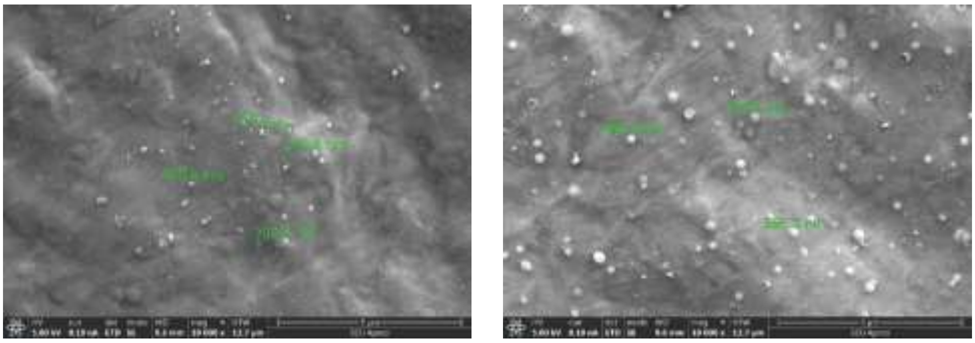

(a) (b)

Figure S5: SEM images of (a) **AIE-1** and (b) **AIE-2** in 100% THF by drop coast method.

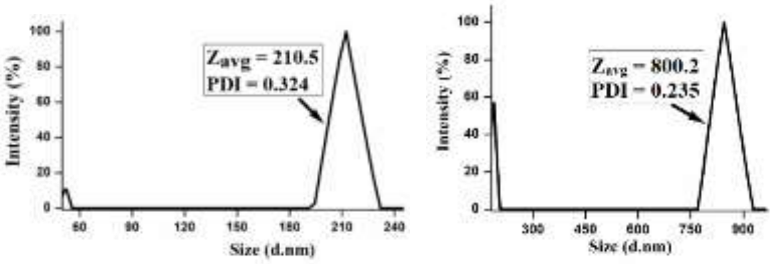

(a) (b)

Figure S6: DLS based particle size analysis of **AIE-2** in (a) 100% THF and (b) THF-H_2O (1:9, v/v)

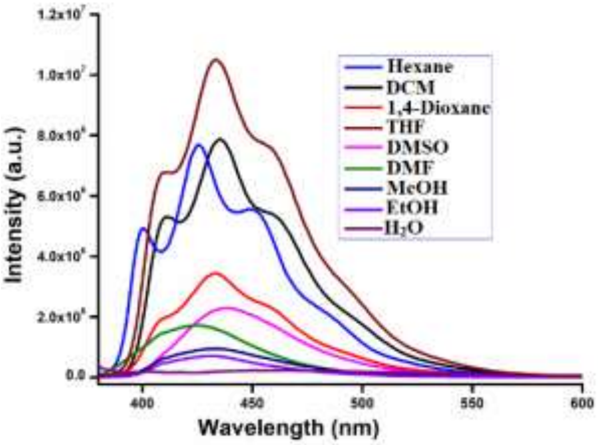

Figure S7: Fluorescence spectra of **AIE-1** in different solvents.

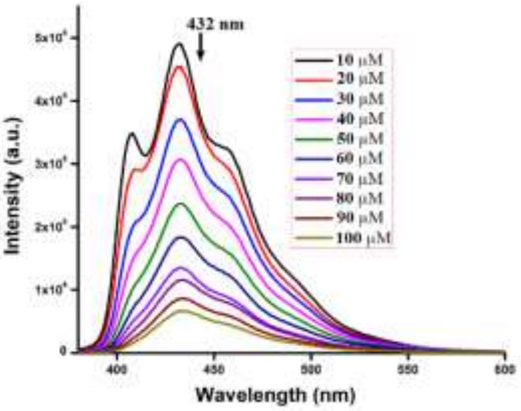

Figure S8: Fluorescence spectra of **AIE-1** in different concentrations.

Under day light

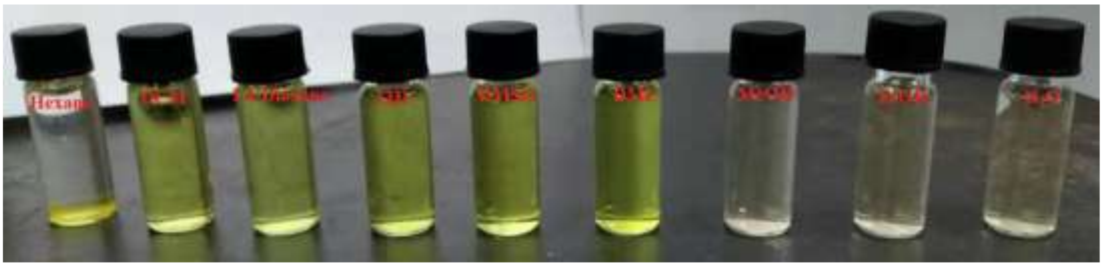

Figure S9: Images of **AIE-2** in different solvents under day light.

Under day light

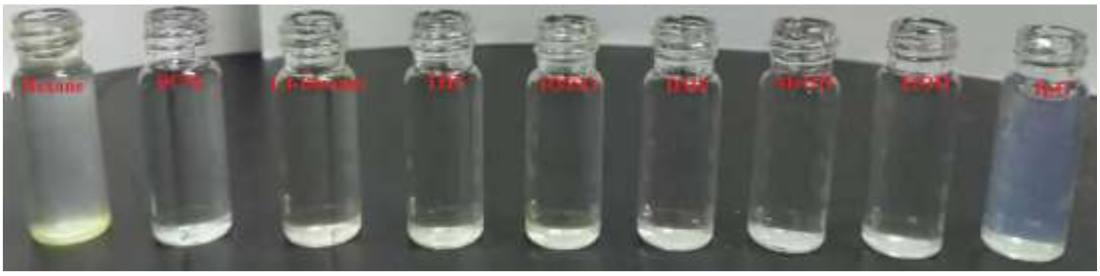

Under 365 nm UV light

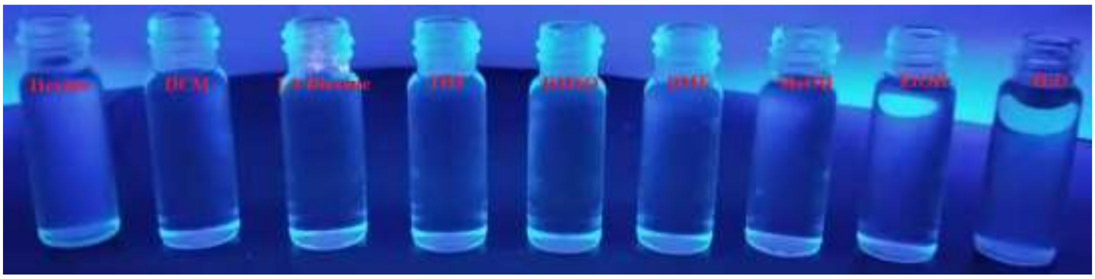

Figure S10: Images of **AIE-1** in different solvents under day and 365 nm UV light.

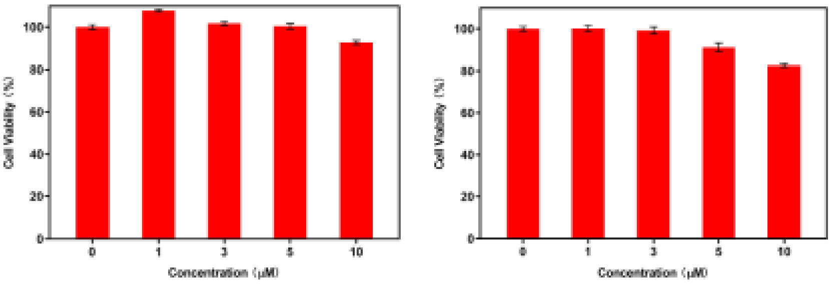

(a) (b)

Figure S11: Cytotoxicity results of (a) **AIE-1** and (b) **AIE-2** on Hela cells using concentrations from 0-10 μM with 48h incubation time.

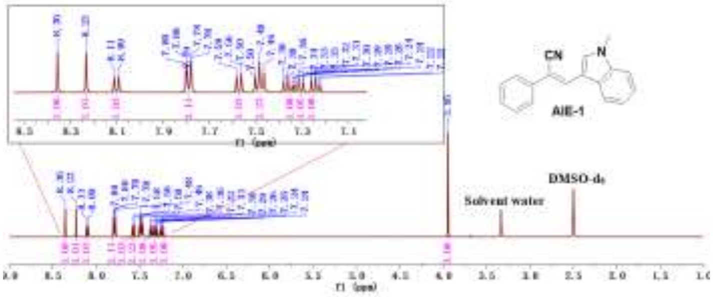

Figure S12: ^{1}H NMR (500 MHz, DMSO-d$_6$) spectra of **AIE-1**.

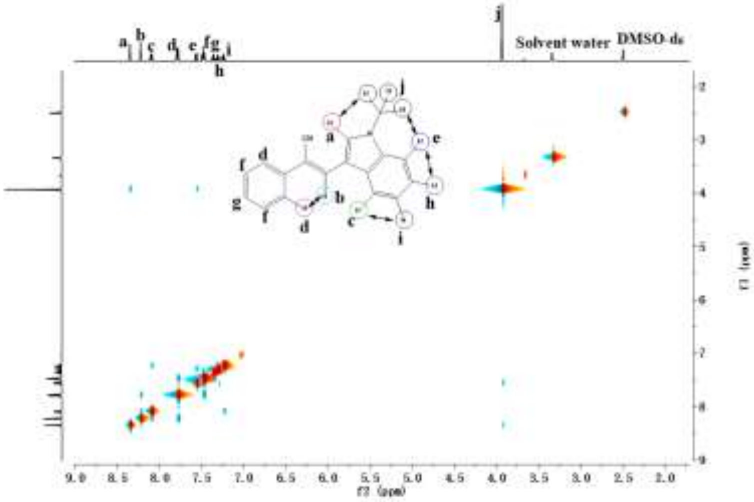

Figure S13: 2D-NOESY (500 MHz, DMSO-d$_6$) spectrum of **AIE-1**.

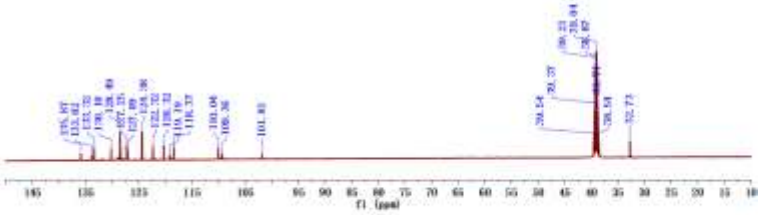

Figure S14: ^{13}C NMR (125 MHz, DMSO-d$_6$) spectra of **AIE-1**.

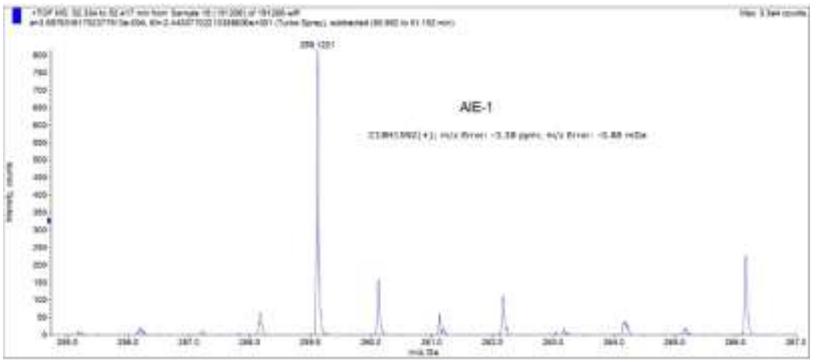

Figure S15: Mass spectrum of **AIE-1**.

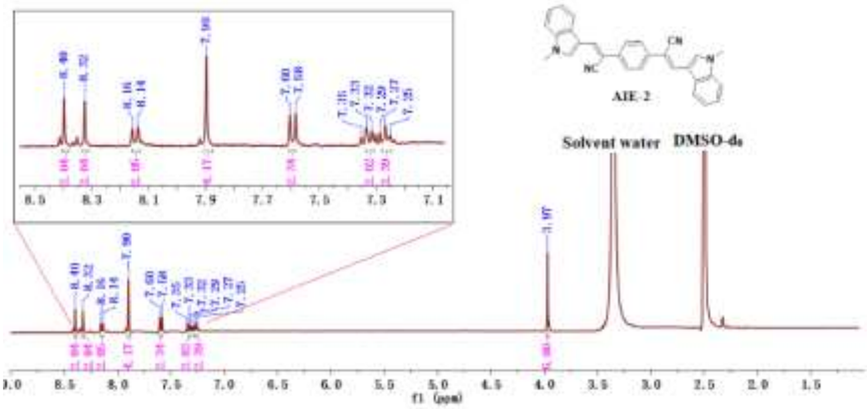

Figure S16: ^{1}H NMR (500 MHz, DMSO-d_6) spectra of **AIE-2**.

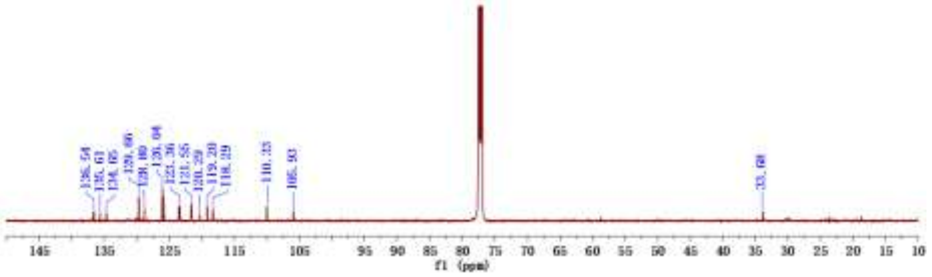

Figure S17: ^{13}C NMR (125 MHz, $CDCl_3$) spectra of **AIE-2**.

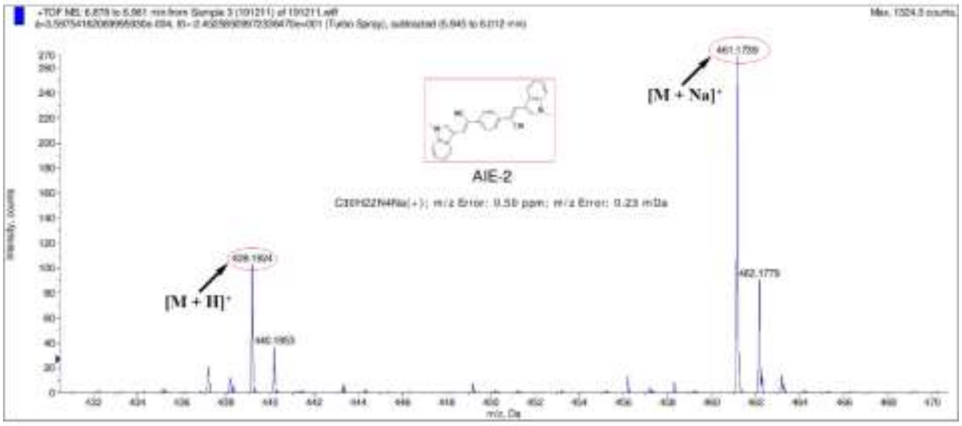

Figure S18: Mass spectrum of **AIE-2**.
